# Differential Expression of S100A Genes in hDPSCs Following Stimulation with Two Hydraulic Calcium Silicate Cements: A Laboratory Investigation

**DOI:** 10.3390/jfb17010055

**Published:** 2026-01-21

**Authors:** Holger Jungbluth, Diana Lalaouni, Jochen Winter, Søren Jepsen, Dominik Kraus

**Affiliations:** 1 Department of Periodontology, Operative and Preventive Dentistry, University Hospital, Faculty of Medicine, University of Bonn, Welschnonnenstr. 17, 53111 Bonn, Germanyjochen.winter@ukbonn.de (J.W.); soeren.jepsen@ukbonn.de (S.J.); 2 Department of Prosthodontics, Preclinical Education and Material Sciences, University Hospital, Faculty of Medicine, University of Bonn, Welschnonnenstr. 17, 53111 Bonn, Germany; dominik.kraus@ukbonn.de

**Keywords:** hydraulic calcium silicate cements, S100A proteins, vital pulp therapy, stem cells

## Abstract

Hydraulic calcium silicate cements (HCSCs) are contemporary materials in vital pulp therapy (VPT) and regenerative endodontic therapy (RET) due to their favorable effects on pulpal and periodontal cells, including cell differentiation and hard tissue formation. Recent studies also indicated the involvement of several S100A proteins in inflammatory, differentiation, and mineralization processes of the pulp. The aim of the present study was to investigate the effects of HCSCs on S100A gene expression in human dental pulp stem cells (hDPSCs). Human DPSCs were isolated and characterized by multi-lineage stem-cell markers and differentiation protocols. In stimulation experiments hDPSCs were exposed to ProRoot^®^MTA, Biodentine^®^, IL-1β, and dexamethasone. Cell viability was determined by XTT assay. IL-6 and IL-8 mRNA expression was measured to analyze proinflammatory response. In addition, odontogenic differentiation and biomineralization assays were conducted (DSPP- and ALP-mRNA expression, ALP activity, and Alizarin Red staining). Differential expression of 13 S100A genes was examined using qPCR. Low concentrations of HCSCs enhanced the proliferation of hDPSCs, whereas higher concentrations exhibited cytotoxic effects. HCSCs induced a pro-inflammatory response and led to odontogenic differentiation and biomineralization. This was accompanied by significant alterations in the expression levels of various S100A genes. ProRoot^®^MTA and Biodentine^®^ significantly affect the expression of several S100A genes in hDPSCs, supporting their role in inflammation, differentiation, and mineralization. These findings indicate a link between the effects of HCSCs on human pulp cells during VPT or RET and S100A proteins.

## 1. Introduction

Since their introduction into modern dentistry in the early 1990s, hydraulic calcium silicate cements (HCSCs) have significantly contributed to the spectrum, success, and predictability of various treatment procedures in endodontics [1]. The two main application areas are vital pulp therapies (VPT), in which the biomaterial is typically applied directly to the vital pulp, and the sealing of the endodontium from the periodontium [1,2,3,4,5]. This includes perforation repair, apexification in immature teeth, and retrograde root filling in the context of apical surgery. Furthermore, there are some special indications where hydraulic calcium silicate cements are also used, such as revitalization treatments [6] and the treatment of invasive cervical resorptions [7]. A comprehensive review on HCSCs categorizes different products based on their intended application: intra-coronal, intra-radicular, or extra-radicular [8].

The generally acknowledged advantage of HCSCs lies in their excellent biocompatibility and bioactivity. For instance, when used in contact with the periodontium, in most cases, the formation of root cement on the materials can be observed [9]. Also, cementoblasts have been shown to grow on HCSC [10], a property that likely contributes to its more biologically favorable healing characteristics compared to conventional materials.

Within the context of VPT, particular attention is given to the characteristics of HCSCs in their interaction with dental pulp cells. Here, the influence of HCSCs on odontoblasts, pulpal fibroblasts, undifferentiated mesenchymal cells, and pulpal stem cells is of significant interest. Nair et al. demonstrated that placement of mineral trioxide aggregate (MTA) to an amputated pulp stump led to the formation of a superior hard tissue barrier compared to the application of a calcium hydroxide cement [11], and other researchers came to comparable results [12,13,14].

In several in vitro studies using human dental pulp cells (hDPCs), MTA was shown to have a good biocompatibility [15,16,17,18,19] and to regulate genes related to signal transduction, protein metabolism, immunity and defense, as well as developmental processes like cell proliferation and differentiation [20,21,22].

Some studies using stem cells of the human dental pulp (hDPSCs) demonstrated that MTA stimulates odontogenic differentiation, proliferation and mineralization [19,23,24,25,26,27,28].

S100 proteins are calcium-binding proteins that belong to a family with at least twenty-one known members [29]. They are involved in numerous cellular processes, including intercellular communication, differentiation, proliferation, and inflammation [30,31,32]. They fulfill diverse functions in various cell types across different tissues and also play a significant role in multiple diseases. These include cancer, cardiovascular, neurological, and chronic inflammatory diseases, as well as developmental disorders. In some disorders, they are used as biomarkers in clinical diagnostics [33].

There is emerging evidence regarding their occurrence and potential functions in the dental pulp. Several authors have described S100A proteins in relation to inflammatory, differentiation, and mineralization processes of the pulp. For example, altered expression levels of S100A genes, as well as changes in the presence of S100A proteins, have been reported in inflamed human dental pulp tissue and in pulps from cariously affected teeth [34,35,36,37].

The proteins S100A7 and -A8 were shown in an animal experiment to promote pulpal wound healing [38]. When used as pulp-capping agents in the same study, they were even able to induce well-structured dentine bridge formation. In a subsequent project, shared functional peptides in the amino acid sequence from the proteins S100A7, -A8, and -A9 were isolated and tested as capping materials. One variant of the tested peptides showed statistically comparable results in dentine bridge formation to the positive control MTA [39]. Other researchers identified S100A7 to be overexpressed in orthodontically treated teeth and hDPCs exposed to mechanical stress, while S100A4 and -A8 expression were unaffected. They also showed that the S100A7 protein promotes osteoclast/odontoclast differentiation as well as their dentine-resorbing activity [40]. Gallorini et al. aimed to identify specific markers of cells at the dentin–pulp interface and described protein S100A4 as a relevant candidate marker for cells of the odontoblast lineage [41]. This protein was also detected in an immunohistochemical analysis of S100A proteins in human pulp tissue at the immediate periphery of interstitial pulpal calcifications and pulp stones [35]. Thus, it can be concluded that they are strongly expressed in hard-tissue-forming cells of the dental pulp.

To date, these findings represent the key insights into S100A proteins in the context of the formative, defensive, and regenerative functions of the dental pulp and its cells. Since HCSCs induce cell differentiation and hard tissue formation, typically accompanied by at least mild inflammation upon contact with pulpal tissue or with hDPCs/hDPSCs, the role of S100A proteins in this context is of particular interest.

However, the influence of HCSCs on the expression of S100A proteins has not yet been investigated.

Therefore, the present study aims to investigate the influence of two commonly used HCSCs on human dental pulp stem cells (hDPSCs). Specifically, the expression of S100A genes, the alteration of mineralization properties, and the inflammatory-chemotactic state of the cells will be examined.

The null hypothesis is that HCSCs do not influence the mineralization properties and the expression of S100A genes in hDPSCs.

## 2. Materials and Methods

### 2.1. Cell Isolation and Characterization

The study was approved by the Ethics Committee of the University of Bonn (Ref. #217/16). Pulpal tissue was harvested from healthy, caries-free teeth that had not yet completed root formation. Tooth extractions had been performed for orthodontic purposes in patients aged 9 to 12 years. Written informed consent was obtained from the parents of the participants for inclusion in the study. Human dental pulp stem cells (hDPSCs) were isolated and cultured using an outgrowth method, as previously described [42]. The cell culture medium consisted of Dulbecco’s Modified Eagle’s Medium (DMEM; Gibco™ DMEM, Fisher Scientific GmbH, Schwerte, Germany), supplemented with 10% fetal bovine serum (FBS), 1% antibiotic and antimycotic solution (both from Gibco™, Fisher Scientific GmbH, Schwerte, Germany), and 5 ng/mL fibroblast growth factor-2 (FGF-2; R&D Systems, Wiesbaden, Germany) to support the maintenance of stem cell properties in the human dental pulp cells [43]. Cells and explants were incubated at 37 °C in a humidified atmosphere with 5% CO_2_, and the culture medium was replaced every 2–3 days. After one week, a sufficient number of cells had migrated from the pulpal tissue, and STRO-1–expressing cells were enriched by magnetic-activated cell sorting (MACS^®^; Miltenyi Biotec, Bergisch Gladbach, Germany) using a mouse monoclonal antibody against STRO-1 (R&D Systems) in accordance with the manufacturer’s protocol. Cells were subsequently cultured in FGF-2-supplemented growth medium and expanded up to the ninth passage. Routine screening for mycoplasma contamination was performed by polymerase chain reaction (PCR) analysis in combination with 4′,6-diamidino-2-phenylindole (DAPI; Sigma-Aldrich, Munich, Germany) staining. The mesenchymal stem cell characteristics of human dental pulp stem cells (hDPSCs) were verified by analyzing the expression of established stem cell markers (CD44, STRO-1, Nestin) and their ability to differentiate into adipocyte-like, chondrocyte-like, osteoblast-like, and odontoblast-like phenotypes through immunofluorescence and immunohistochemical staining.

### 2.2. Immunofluorescence Microscopy

Cells were seeded onto sterile coverslips and cultured for 24 h, followed by fixation with 4% paraformaldehyde (Sigma-Aldrich) for 15 min. The cells were then washed with phosphate-buffered saline (PBS) and permeabilized with 0.1% Triton X-100 (Sigma-Aldrich) in PBS for 15 min. After additional PBS washes, cells were blocked with 5% goat serum (DAKO, Hamburg, Germany) for 1 h at room temperature (RT) and subsequently incubated overnight at 4 °C with primary antibodies against CD44 (DAKO, 1:50), Nestin (DAKO, 1:100), Stro-1 (R&D Systems, 1:50), DSPP (Bioss, Woburn, MA, USA, 1:100), and NFκB p65 (Cell Signaling Technologies, Leiden, Netherlands, 1:200) in Tris-buffered saline (TBS) containing 1% bovine serum albumin (BSA, Sigma-Aldrich). Following extensive PBS washes, cells were incubated with a Cy3- or Alexa Fluor-488-conjugated secondary antibody (Dianova, Hamburg, Germany) at a 1:250 dilution for 1 h at RT. After another round of PBS washing, nuclear staining was performed using 4′,6-diamidino-2-phenylindole (DAPI; Sigma-Aldrich) for 8 min. The coverslips were then washed with PBS and mounted on glass slides using Mowiol/DABCO (Roth, Karlsruhe, Germany) for fluorescence microscopy analysis (Axio Imager.A1 microscope with Axiomcam 305 color and Zeiss Zen Version 3.11; Carl Zeiss Microscopy Deutschland GmbH, Oberkochen, Germany).

### 2.3. Cell Differentiation

To verify the stem cell characteristics of the isolated human dental pulp stem cells (hDPSCs), we applied various cell differentiation models as previously described [42]. Cells were seeded onto 12-well plates at an initial density of 50,000 cells per well and cultured until 100% confluence was reached. Differentiation protocols were then applied to induce adipogenic, osteogenic, odontogenic, and chondrogenic differentiation.

For adipogenic differentiation, hDPSCs were cultured in adipogenesis-inducing medium for 5 days, starting 2 days after reaching confluence. The medium consisted of DMEM (4.5 g/L glucose), 10% FBS, 1% antibiotic and antimycotic solution (Gibco™; Fisher Scientific GmbH, Schwerte, Germany), and was supplemented with 1 µM dexamethasone, 0.2 mM indomethacin, 1.7 µM insulin, and 0.5 mM 3-isobutyl-1-methylxanthine (all from Sigma-Aldrich). This was followed by a 2-day incubation in adipogenesis maintenance medium (DMEM, 4.5 g/L glucose, 1.7 µM insulin, 10% FBS, 1% antibiotic and antimycotic solution). The differentiation protocol was repeated twice for a total period of 21 days.

Osteogenic and odontogenic differentiation was induced by incubating confluent hDPSCs in osteogenic medium for 30 days, with medium changes every third day. The osteogenic medium contained DMEM (4.5 g/L glucose), 10% FBS, 1% antibiotic and antimycotic solution (Gibco™), 0.2 mM L-ascorbic acid 2-phosphate, 10 nM β-glycerophosphate, and 100 nM dexamethasone (all from Sigma-Aldrich).

Chondrogenic lineage commitment was achieved using a three-dimensional pellet culture system. Briefly, 2 × 10^6^ cells were collected and pelleted by centrifugation at 500× *g* in 15 mL polypropylene conical tubes, after which the pellets were maintained in culture for a period of four weeks. Chondrogenic induction was performed in a serum-free, chemically defined medium based on high-glucose DMEM (4.5 g/L; Gibco™) and supplemented with insulin (6.25 µg/mL), transferrin (6.25 µg/mL), selenous acid (6.25 µg/mL), linoleic acid (5.33 µg/mL), bovine serum albumin (1.25 µg/mL), sodium pyruvate (1 mM), L-ascorbic acid 2-phosphate (0.1 mM), dexamethasone (100 nM; all from Sigma-Aldrich), and transforming growth factor beta 3 (TGF-β3; 10 ng/mL; R&D Systems). Cultures were maintained at 37 °C under humidified conditions with 5% CO_2_, and the differentiation medium was refreshed every two to three days.

Differentiation was assessed using specific staining methods: adipogenic differentiation was evaluated by Oil Red O staining, osteogenic differentiation by Alizarin Red staining, odontogenic differentiation by DSPP staining, and chondrogenic differentiation by Toluidine Blue and Collagen-II staining.

#### 2.3.1. Oil Red O Staining

Cells on sterile coverslips were washed twice with PBS and then fixed with 4% paraformaldehyde (Sigma-Aldrich) for 10 min at room temperature (RT). After fixation, cells were rinsed with 50% ethanol (AppliChem, Darmstadt, Germany). Oil Red O staining was then performed for 30 min, followed by washing with 50% ethanol and distilled water. Nuclear staining was conducted using Mayer’s hematoxylin (Merck, Darmstadt, Germany) for 2 min, followed by a 5 min wash with running water. Finally, the coverslips were mounted using Aquatex^®^ (Merck).

#### 2.3.2. Alizarin Red Staining

Following a modified protocol by Gregory et al. [44], cells were washed twice with PBS and fixed with 4% paraformaldehyde (Sigma-Aldrich) for 20 min at room temperature (RT). After fixation, cells were rinsed with distilled water and incubated with a 40 mM Alizarin Red solution (Sigma-Aldrich) for 20 min at pH 4.1. Cells were then washed five times with distilled water and mounted with Aquatex^®^.

#### 2.3.3. Toluidin Blue Staining

To verify chondrogenic differentiation, cryosections of cell pellets were prepared and stained with Toluidine Blue solution (Sigma-Aldrich) for 3 min. After staining, sections were washed with running water and mounted with Aquatex^®^.

#### 2.3.4. Collagen-II Staining

Sections of cell pellets were fixed with 100% methanol (AppliChem) for 8 min at −20 °C. After fixation, the sections were rehydrated with PBS containing 0.1% Triton X-100 (Sigma-Aldrich) for 10 min. Pellets were then treated with hyaluronidase (Sigma-Aldrich) for 30 min at room temperature (RT). Following this, sections were washed with distilled water and Tris-buffered saline (TBS), and incubated with proteinase K (DAKO) for 30 min. After another wash with TBS, unspecific binding sites were blocked by incubating the sections with 10% anti-goat serum (DAKO) in TBS for 1 h at RT.

Subsequently, sections were first rinsed once in Tris-buffered saline (TBS) and then incubated with an anti-collagen II primary antibody (Acris Antibodies, Hiddenhausen, Germany) at 4 °C overnight. Following thorough washing steps in phosphate-buffered saline (PBS), a Cy3-labeled goat anti-rabbit IgG secondary antibody (Dianova, Hamburg, Germany) was applied at a dilution of 1:250 for 1 h at room temperature. After additional PBS washes, nuclei were counterstained with 4′,6-diamidino-2-phenylindole (DAPI; Sigma-Aldrich) for 5 min. The sections were then rinsed again in PBS and mounted onto glass slides using Mowiol/DABCO mounting medium (Roth, Karlsruhe, Germany).

### 2.4. Hydraulic Calcium Silicate Cement Preparation/Preparation of Conditioned Media

The HCSCs MTA (ProRoot^®^ MTA white; Dentsply Tulsa Dental) and Biodentine^®^ (Septodont GmbH, Niederkassel, Germany) were prepared and molded into disks with a diameter of 15 mm and a thickness of 1.5 mm under aseptic conditions, following the manufacturers’ instructions. The cement discs were incubated for 24 h at 37 °C in a humidified atmosphere containing 5% CO_2_. After incubation, the materials were sterilized using ultraviolet (UV) light for 15 min.

Once the cements had fully set, the discs were placed in a mortar and ground into powder using a pestle under sterile conditions. A 20 mg/mL concentration of each powder was then dissolved in DMEM, mixed using a vortex, and stored for 24 h in an incubator at 37 °C with 100% humidity and 5% CO_2_. The resulting conditioned media were centrifuged at 45× *g* for 5 min at 4 °C. The supernatants were filtered through a Millex^®^ syringe filter with a 0.45 µm pore size (EMD Millipore Corporation, Billerica, MA, USA) and further diluted in DMEM as needed, based on the 20 mg/mL stock solutions. The conditioned media were used for stimulation experiments and were freshly prepared for each set of experiments.

### 2.5. Assessment of Cell Viability Using XTT Assay

Cell viability was assessed using the PromoKine XTT Assay Kit (Promocell, Heidelberg, Germany). In brief, 10,000 hDPSCs (third passage) were seeded per well in 96-well plates and incubated for 24 h. To minimize evaporation effects in the peripheral wells, cells were cultured only in the central wells, while the outer wells were filled with sterile water. The cells were then exposed to the two conditioned HCSC media at varying concentrations (20, 10, 2, 0.2, 0.02, and 0.002 mg/mL). After 24 h of incubation, XTT reaction solution was added to the medium and incubated for 3 h. Absorbance was measured at 490 nm with a correction wavelength of 670 nm using a microplate reader. Cell culture medium without additives served as the control. Cell viability was calculated by normalizing the absorbance values to the control (100%). Experiments were conducted with hDPSCs from three different donors, with each condition tested in hexaduplicate.

### 2.6. Cell Stimulation Exposure

For stimulation experiments, third-passage cells from two different donors were seeded in triplicates on 12-well plates at an initial density of 50,000 cells per well. Once cells reached 90% confluence, they were cultured under serum-free conditions for 24 h. Subsequently, the dental pulp stem cells were stimulated with the two conditioned HCSC media at concentrations of 0.002, 0.02, and 0.2 mg/mL, which were selected based on the viability results. Two additional groups were treated with dexamethasone (100 nM; Sigma-Aldrich) as an inducer of osteogenic differentiation and with human interleukin-1β (IL-1β; 10 ng/mL; R&D Systems) to simulate a pro-inflammatory environment. Cells without stimulation served as control. After 24 and 72 h, cells were collected for mRNA expression analysis.

### 2.7. RNA-Isolation, First Strand cDNA Synthesis, qPCR

Total RNA was extracted using the RNeasy Plus Mini Kit (Qiagen, Hilden, Germany) in accordance with the manufacturer’s instructions. RNA concentration and purity were assessed with a NanoDrop ND-1000 spectrophotometer (NanoDrop Technologies, Wilmington, DE, USA). For complementary DNA (cDNA) synthesis, 1 µg of total RNA was reverse transcribed using the iScript™ Select cDNA Synthesis Kit (Bio-Rad Laboratories, Munich, Germany) with oligo(dT) primers. Real-time PCR experiments were carried out according to “MIQE Guidelines: Minimum Information for Publication of Quantitative Real-Time PCR Experiments” [45] and described recently [34,46]. The expression levels of IL6, IL8, ALP, DSPP, and thirteen S100A genes (S100A1, -A2, -A3, -A4, -A6, -A7, -A8, -A9, -A10, -A11, -A13, -A14, and -A16) were quantified by quantitative polymerase chain reaction (qPCR). Primer sequences, their annealing temperatures and efficiencies, and the qPCR conditions have been described previously [IL6, IL8, DSPP: [42] and S100 genes: [34]].

### 2.8. Assessment of Osteogenic Differentiation Using Alkaline Phosphatase (ALP) Activity Assay and Alizarin Red Staining (ARS)/CPC Extraction Assay

For the determination of alkaline phosphatase (ALP) activity and biomineralization (ARS/CPC extraction), dental pulp stem cells from three different donors were seeded in triplicates on 12-well plates. Upon reaching confluency, hDPSCs were cultured in osteogenic medium containing 0.2 mM L-ascorbic acid 2-phosphate and 10 nM β-glycerophosphate (Sigma-Aldrich). Cells were then stimulated with HCSC-conditioned media at varying concentrations (0.2, 0.02, and 0.002 mg/mL), dexamethasone (100 nM), or IL-1β (10 ng/mL; 0.6 nM) for either 6 days (for ALP activity) or 30 days (for ARS/CPC). The medium was changed every three days. Cells cultured in osteogenic medium alone served as the control.

ALP enzyme activity in dental pulp stem cell lysates was determined by measuring the hydrolysis of para-nitrophenylphosphate, as described elsewhere [47]. The samples were quantified by photometric analysis at a wavelength of 562 nm. Total protein concentration was determined using the Bicinchoninic Acid Protein Assay (Thermo Fisher Scientific, Bonn, Germany), with bovine serum albumin serving as the standard. Specific ALP activity was calculated as the amount of p-nitrophenyl formed per minute, normalized to the total protein concentration and expressed relative to the control group.

The biomineralization capacity of hDPSCs was assessed by staining calcium deposits with Alizarin Red solution (ARS). After 30 days of stimulation, cells were washed twice with PBS and fixed in 4% paraformaldehyde for 30 min at room temperature. After two rinses with distilled water (dH_2_O), the cells were exposed to a 40 mM Alizarin Red S (ARS) solution (pH 4.1; Sigma-Aldrich, Munich, Germany) and incubated for 30 min under gentle agitation. Subsequently, the staining reagent was discarded, and mineralized extracellular matrices were thoroughly rinsed five times with dH_2_O to remove excess dye. For quantitative dye extraction, 10% (*w*/*v*) cetylpyridinium chloride (CPC) prepared in 10 mM sodium phosphate buffer (pH 7.0) was added to each well, followed by incubation with mild agitation for an additional 30 min. The eluates were transferred to 1.5 mL reaction tubes, centrifuged, and the optical density of the supernatants was measured at 562 nm.

### 2.9. Statistical Analysis

All statistical evaluations were conducted using GraphPad Prism (version 10; GraphPad Software, San Diego, CA, USA). Experimental results are reported as mean values with the corresponding standard error of the mean (SEM). Group comparisons were performed using one-way analysis of variance (ANOVA), followed by Dunnett’s multiple comparisons test. Differences were considered statistically significant when *p* values were below 0.05.

## 3. Results

### 3.1. Cell Characterization

**Figure 1:**
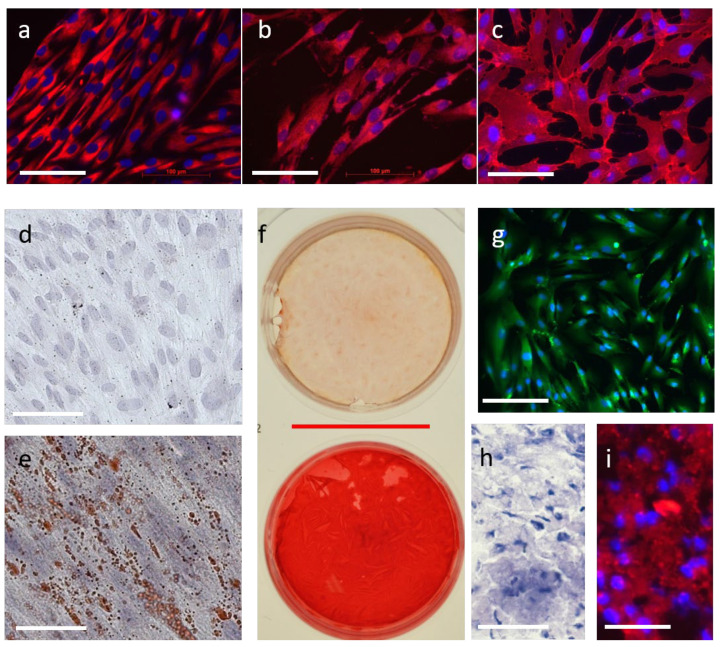
Cell characterization of human dental pulp stem cells (hDPSCs). Immunofluorescence staining of hDPSCs revealed positive expression for Stro-1 (**a**), Nestin (**b**), and CD44 (**c**). Vizualization of various differentiation and staining protocols (**d**–**i**). Adipocyte differentiation was verified by Oil red O staining showing characteristic lipid packed vacuoles in the cytoplasm (red) (**e**) compared to undifferentiated control cells (**d**). Osteoblastic-like (**f**) and odontoblast-like (**g**) phenotype was affirmed by Alizarin Red ((**f**); upper well: control cells, lower well: stimulated cells) and DSPP staining (**g**). Chondrocyte differentiation was confirmed by Toluidin blue ((**h**); glycosaminoglycans are stained blue) and Collagen II (red) staining (**i**). White scale bars indicate 100 µm, whereas the red scale bar indicates 500 µm.

Isolated Stro-1-positive dental pulp cells were characterized for the expression of mesenchymal and neural stem cell markers, as well as their differentiation potential. Immunofluorescence staining revealed that all cells were positive for CD44, Nestin, and Stro-1 (Figure 1a–c). Nestin (Figure 1a) and Stro-1 (Figure 1b) were mainly detected in the cytoplasm, whereas CD44 (Figure 1c) was predominantly localized at the cell membrane. Furthermore, the isolated dental pulp stem cells successfully differentiated into adipocyte-, chondrocyte-, osteoblast-, and odontoblast-like phenotypes, as demonstrated by specific differentiation protocols and histological staining (Figure 1d–i). Adipogenic differentiation was confirmed by Oil Red O staining, showing characteristic cytoplasmic lipid-laden vacuoles (red staining; Figure 1e) compared to undifferentiated control cells (Figure 1d). Chondrogenic differentiation was evidenced by Toluidine Blue staining (Figure 1h) and the expression of Collagen II (red staining; Figure 1i). Differentiation toward an osteoblast-like phenotype was validated by Alizarin Red staining (Figure 1f): incubation with osteogenic medium markedly increased extracellular calcium deposits, visible as an intense red staining, in contrast to the control cells. In addition, odontogenic differentiation was indicated by cytoplasmic expression of DSPP (green staining; Figure 1g). Collectively, these findings demonstrate that Stro-1-positive dental pulp stem cells express both mesenchymal and neural stem cell markers and possess a broad multi-lineage differentiation potential.

### 3.2. Cell Viability

**Figure 2:**
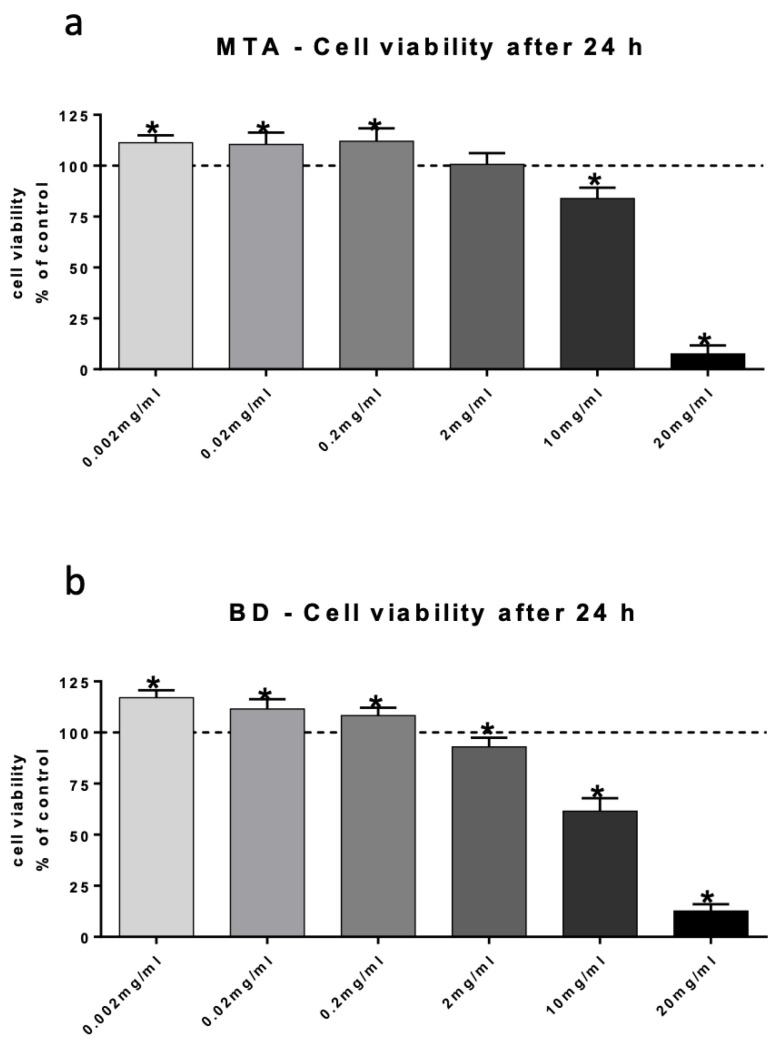
Cell viability analysis. Cell viability of hDPSCs after stimulation with ProRoot^®^MTA (**a**) and Biodentine^®^ (**b**) for 24 h using XTT assay. Unstimulated cells served as control. Data are presented as mean ± SEM. Statistical analysis was performed using one-way ANOVA followed by Dunnett’s post hoc test (* *p* < 0.05).

Results of hDPSC viability assessment following incubation with different concentrations of ProRoot^®^MTA and Biodentine^®^ are depicted in Figure 2. For both HCSCs the lowest concentrations (0.002, 0.02, and 0.2 mg/mL) resulted in enhanced cell proliferation when compared with unstimulated control. At increasing concentrations, however, cell proliferation stagnated, and a cytotoxic effect was observed at highest concentrations.

### 3.3. Inflammatory Response of hDPSCs Following Stimulation with ProRoot^®^MTA and Biodentine^®^

**Figure 3:**
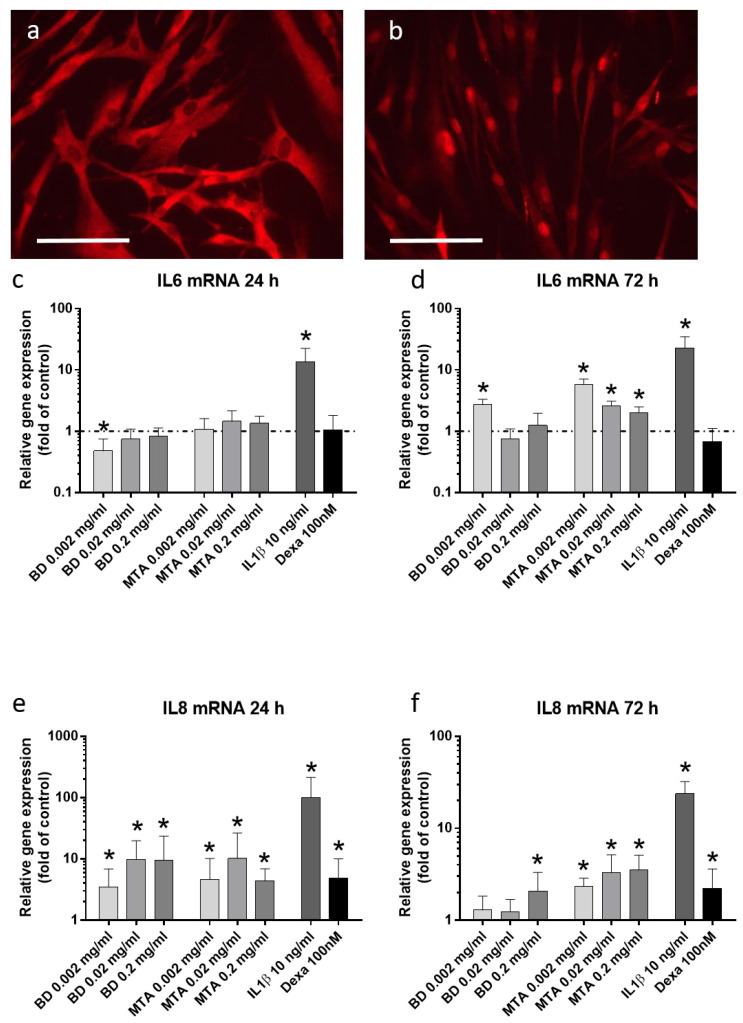
Inflammatory response of hDPSCs following stimulation experiments. Analysis of inflammatory processes in hDPSCs was performed by the visualization of NF-κΒ translocation after stimulation with IL-1β (10 ng/mL) for 2 h (**b**). Unstimulated cells served as control (**a**). White scale bars indicate 100 µm. mRNA expression of IL-6 ((**c**): 24 h and (**d**): 72 h) and IL-8 ((**e**): 24 h and (**f**): 72 h) following stimulation with Biodentine^®^ (BD), ProRoot^®^MTA (MTA), IL-1β, and Dexamethasone (Dexa). Data are presented as mean ± SEM. Statistical analysis was performed using one-way ANOVA followed by Dunnett’s post hoc test (* *p* < 0.05).

Expression of IL-6 and IL-8 following stimulation of hDPSCs for 24 and 72 h with conditioned media of HCSC are presented in Figure 3. Both tested HCSCs provoked an inflammatory response in the cells. IL-6 expression was significantly increased only after 72 h, while IL-8 expression was increased already after 24 h. Lowest concentrations of both, ProRoot^®^MTA and Biodentine^®^, led to the highest expression levels of IL-6 after 72 h (5.7 +/− 1.4-fold and 2.7 +/− 0.6-fold). Nuclear translocation of nuclear factor κΒ (NF-κΒ) due to stimulation with IL-1β in the isolated stem cells was demonstrated during characterization (cf. Figure 3b).

### 3.4. Osteogenic/Odontogenic Differentiation of hDPSCs

**Figure 4:**
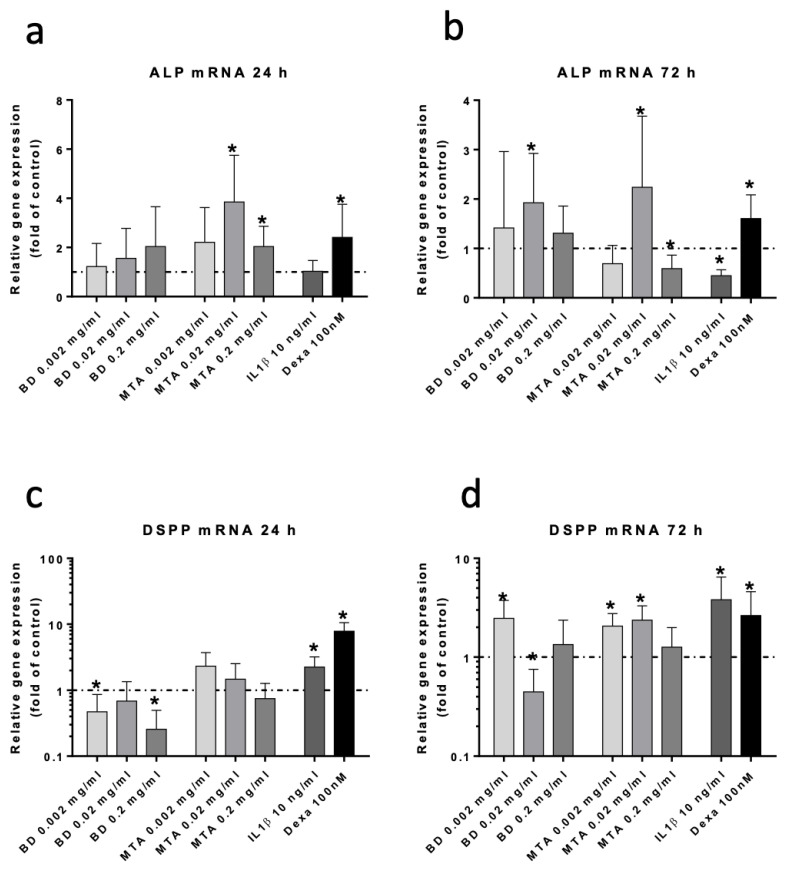
Expression of osteogenic/odontogenic differentiation markers. mRNA expression of ALP ((**a**): 24 h and (**b**): 72 h) and DSPP ((**c**): 24 h and (**d**): 72 h) following stimulation with Biodentine^®^ (BD), ProRoot^®^MTA (MTA), IL-1β, and Dexamethasone (Dexa). Data are presented as mean ± SEM. Statistical analysis was performed using one-way ANOVA followed by Dunnett’s post hoc test (* *p* < 0.05).

**Figure 5:**
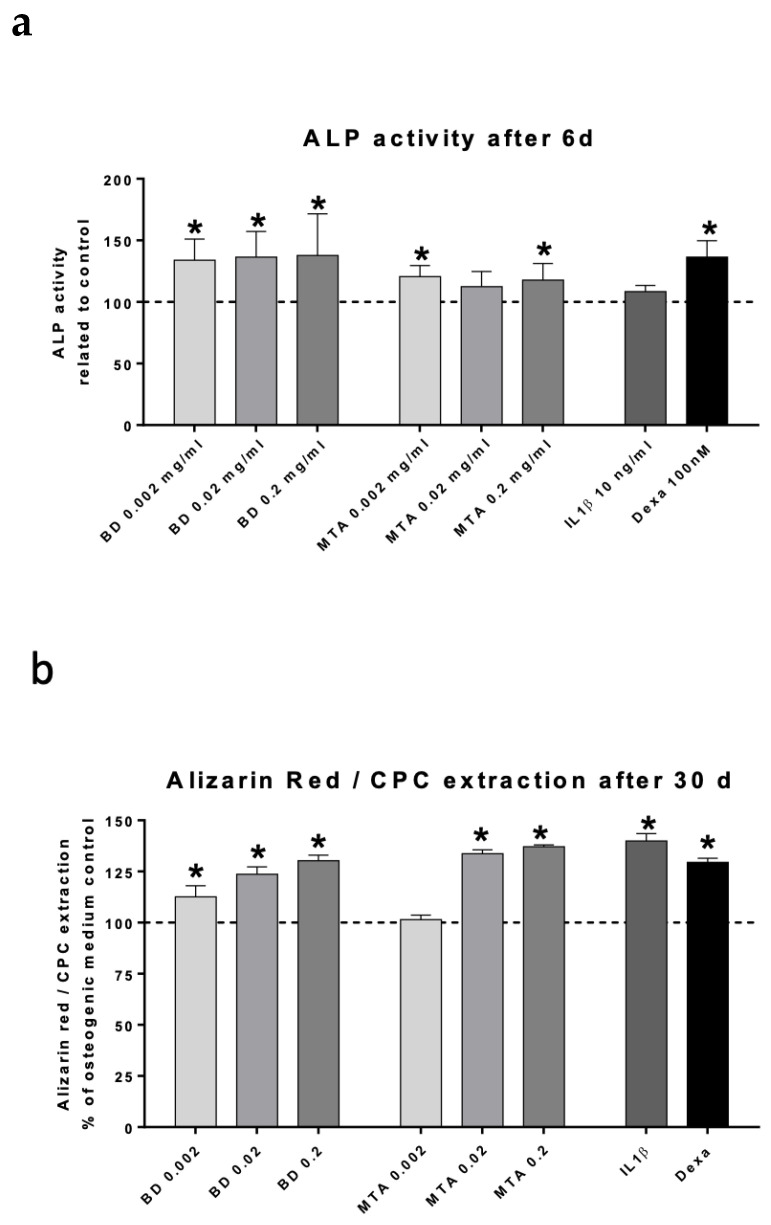
Analysis of alkaline phosphatase (ALP) activity and biomineralization. ALP activity in hDPSCs following stimulation with Biodentine^®^ (BD), ProRoot^®^MTA (MTA), IL-1β, and Dexamethasone (Dexa) after 6 d (**a**). Specific ALP activity was calculated as the amount of p-nitrophenyl formed per minute, normalized to the total protein concentration and expressed relative to the control cells. Biomineralization capacity was assessed by staining calcium deposits with Alizarin Red staining and CPC extraction after 30 days (**b**). Optical densities were normalized to those from control cells. Data are presented as mean ± SEM. Statistical analysis was performed using one-way ANOVA followed by Dunnett’s post hoc test (* *p* < 0.05).

Alkaline phosphatase (ALP) and dentin sialophosphoprotein (DSPP) expression are shown in Figure 4a and Figure 4b, respectively. ALP activity and Alizarin Red/CPC extraction are presented in Figure 5a and Figure 5b, respectively.

Expression of ALP was significantly increased after 24 h following stimulation with 0.02 and 0.2 mg/mL MTA. After 72 h, stimulation with 0.02 mg/mL MTA and Biodentine^®^ led to significant ALP overexpression, while 0.2 mg/mL MTA led to decreased ALP expression.

Expression of DSPP was increased only after 72 h of stimulation with 0.002 mg/mL Biodentine^®^ and with 0.002 and 0.02 mg/mL MTA. Reduced expression of DSPP was observed following 24 h of stimulation with Biodentine^®^ at concentrations of 0.002 and 0.2 mg/mL, and after 72 h with a concentration of 0.02 mg/mL.

ALP activity after 6 d increased to the level of dexamethasone (positive control) in all three concentrations of Biodentine^®^ (0.002, 0.02, and 0.2 mg/mL; about 135% +/− 24%), while MTA promoted ALP activity slightly less (about 117% +/− 11%).

Alizarin Red staining was significantly increased in all concentrations of both HCSCs with exception of the lowest concentration of MTA (0.002 mg/mL). Biodentine^®^ induced a dose-dependent increase in hard tissue formation, as evidenced by enhanced Alizarin Red staining at higher concentrations.

### 3.5. S100A Gene Expression of hDPSCs

The expression levels of 13 S100A genes following stimulation with three different concentrations of MTA and Biodentine^®^, respectively, are given in Table 1. After 24 h of stimulation, decreased expression levels were found for all three concentrations of MTA and for the lowest concentration of Biodentine^®^ (0.002 mg/mL). The most commonly altered gene expressions were those of S100A1, -A3, -A11, and -A16. S100A1 was the only gene that showed an increased expression after 24 h (after stimulation with 0.2 mg/mL Biodentine^®^).

After 72 h of stimulation, gene expression levels in most cases returned to baseline values or even exhibited a significant upregulation. This effect was particularly evident in the MTA group with the highest concentration (0.2 mg/mL), where genes S100A2, -A3, -A11, -A13, and -A16 turned from initial decrease (24 h) to a marked increase in expression after 72 h. Significant upregulation of gene expression was also detected in the Biodentine^®^ (S100A2, -A4, and -A13) and the MTA group (S100A7) at their lowest tested concentrations (0.002 mg/mL).

No expression of the S100A8 and -A9 genes was detected in any of the test or control groups.

## 4. Discussion

The present study demonstrated that the two commonly used hydraulic calcium silicate cements ProRoot^®^MTA and Biodentine^®^ exert significant effects on the inflammatory response, differentiation, and mineralization capacity, as well as the expression of S100A genes in human dental pulp stem cells. Therefore, the null hypothesis was rejected.

To the best of our knowledge, this is the first report of the differential expression of a large number of S100A genes in well-characterized hDPSCs following stimulation with MTA and Biodentine^®^. Our results demonstrate that the expression of most of the analyzed genes was altered, with the changes being both time- and concentration-dependent with respect to the biomaterial and with variations observed across individual S100A genes. S100A proteins should thus be considered in the context of the physiological processes underlying VPT and RET, and in the development and refinement of associated biomaterials and techniques.

We first performed cell viability assays and demonstrated that the growth of hDPSCs is positively influenced by low concentrations of the two applied HCSCs. However, at concentrations exceeding 2 mg/mL, a cytotoxic effect was observed. This finding is consistent with results from other research groups [15,16,17,19,27]. While the proliferative effect of HCSC on hDPSC is generally acknowledged, various studies yield inconsistent results regarding the quality, quantity, and differences between products. A likely explanation for these discrepancies is the use of differing experimental methodologies. For instance, enhanced cell proliferation was found in studies working with extracts in comparison with those working with direct contact tests [8].

According to our results, stimulation with ProRoot^®^MTA and Biodentine^®^ induced an inflammatory response in hDPSCs. Exposure to the two HCSCs led to transcriptional activation of the inflammation-related genes, IL-6 and IL-8, in the same magnitude as the inflammatory mediator IL-1β.

IL-6 is a pleiotropic cytokine that exerts both pro-inflammatory and anti-inflammatory effects and can modulate the innate immune response through a plethora of immunological pathways [48]. IL-8 is a key player in the recruitment of neutrophils during early stages of immune response but also contributes to angiogenesis, proliferation, and tumor growth [49].

Both IL-6 and IL-8 have been demonstrated to be expressed by hDPCs and odontoblast-like cells stimulated with lipoteichoic acid or a TLR2 agonist [50,51]. The application of MTA, however, reduced IL-6 expression from LTA-stimulated hDPCs [52]. The same effect was observed for IL-8 when hDPCs were stimulated with MTA alone (without LTA), as determined by RNA microarray analysis [20]. Results consistent with ours have been observed in other studies following the application of HCSC on immortal hDPCs [53,54]. Some authors concluded that HCSC has an anti-inflammatory effect, as IL-1α and interferon-γ expressions are reduced following MTA application in murine pulp tissues [55], and the expression of IL-1α and IL-6 was downregulated in murine macrophages [56]. HCSC generally elicited less inflammatory response in comparison with other capping materials, as has been demonstrated in numerous animal studies [8].

In our study IL-6 expression was increased only after 72 h and IL-8 expression after 24 and 72 h. Currently, the exact significance of IL-6 and IL-8 expression following HCSC application cannot be conclusively estimated. Due to the complex regulatory mechanisms involved, the immunomodulatory nature of these two pro-inflammatory cytokines appears to result in varying effects, including both pro-inflammatory and anti-inflammatory responses. It is also plausible that their effects are time-dependent, with an initial pro-inflammatory response followed by a more anti-inflammatory or modulating effect at later stages after HCSC application. The time-dependent response of pulp tissue to the application of HCSC currently is generally accepted [8].

Analysis of DSPP and ALP expression, as well as Alizarin Red staining and ALP activity assays, are commonly used techniques to assess the odontogenic differentiation and mineralization potential of pulp cells. ProRoot^®^MTA and Biodentine^®^ induced significant differentiation as well as mineralization on the hDPSCs. This effect is consistent with the findings of numerous studies in this context [15,16,17,18,19,21,22,24,26,27,57].

Other researchers have already demonstrated that MTA induces odontogenic and osteogenic differentiation in stem cells of the apical papilla (SCAPs) and human periodontal ligament stem cells (hPDLSCs) via the inflammation-related NF-κΒ signaling pathway [58,59]. Thus, the NF-κΒ signaling pathway can be acknowledged as a molecular link between inflammatory and differentiation processes. In our study, the nuclear translocation of NF-κΒ induced by IL-1β and subsequent upregulation of proinflammatory cytokines led also to an odontogenic differentiation shown by upregulated DSPP expression and enhanced extracellular mineralization. In our study, the observed odontogenic differentiation of hDPSCs induced by HCSCs can be attributed, at least in part, to the activation of inflammatory signaling pathways.

Significant alterations in the expression of many S100A genes were observed following stimulation of hDPSCs with the two HCSCs. After 24 h, expression levels were mostly decreased, with a higher number of genes being affected by ProRoot^®^MTA compared to Biodentine^®^. In contrast, after 72 h, increased expression levels predominated.

The method used in the present study does not allow direct conclusions to be drawn about the functions of individual S100A genes in human pulp tissue. However, the altered expression following stimulation with the two HCSCs indicates the involvement of S100A genes in the cellular processes triggered by HCSCs in the hDPSCs. It remains to be clarified which cellular mechanisms underlie this involvement. A possible indication regarding this question is provided when considering the signal transduction pathways that have been described in recent years for both the effects of HCSCs on hDPCs/hDPSCs and those of various S100A proteins, albeit here in the context of other tissues, cell types, and disease contexts.

Chen et al. revealed that odontogenic differentiation, proliferation, and mineralization in hDPCs stimulated by HCSC application is mediated by activation of the Wnt/β-catenin signaling pathway [16]. The Wnt/β-catenin signaling pathway is directly involved in the rate of dentine secretion and has an influence on the quality of the hard tissue formation [60]. Other researchers showed that β-catenin expression is upregulated next to pulp capping with Biodentine^®^ in rat molars [61]. Rathinam confirmed these results and identified by RNA sequencing the upregulation of gene expression of seven gene sets related to repair and regeneration in the dental pulp complex after stimulation with MTA and Biodentine^®^ for 72 h [62]. These included the gene set signatures of TNF-α signaling via NF-κβ, WNT/β-catenin signaling, apoptosis, angiogenesis, inflammatory response, TGF-β signaling, and oxidative phosphorylation [62].

As mentioned above, S100A proteins have been shown to act via these signaling pathways in various tissues and cell types. For example, the following S100A proteins have been shown to regulate specific functions via the Wnt/β-catenin pathway: S100A2 protein promotes epithelial–mesenchymal transition in pulmonary fibrosis [63], S100A4 induces cardiac fibrosis during myocardial ischemic diseases [64] and prostatic stromal cell fibrosis in hyperplastic prostate [65], S100A6 modulates apoptosis of cardiomyocytes [66] and of nucleus pulposis cells [67], S100A8 regulates hair follicle stem cell growth [68], extracellular complex of S100A8/-A9 contributes to colorectal carcinoma cell survival and migration [69], S100A9 and S100A11 stimulate epithelial–mesenchymal transition in cervical cancer cells [70,71], and S100A16 promotes renal fibrosis in acute kidney injury [72].

Additionally, the proteins S100A1, S100A4, the complex S100A8/A9, and S100A9 in its monomeric form regulate inflammatory responses via the TLR4/NF-κΒ signaling pathway in various cell types and tissues, including cardiomyocytes [73], mononuclear cells [74], astrocytes [75], granulocytes [76], pulmonary tissue [77], and nucleus pulposus cells [78].

S100A proteins also modulate cellular and tissue functions through various other signaling pathways such as RAGE, TNF-α, G protein-coupled receptors, CD36, and 5-hydroxytryptamine receptor 1B [79].

The expression of S100 proteins themselves can, on the other hand, be regulated via some of these signaling pathways. For example, S100A4 expression is regulated via the Wnt/β-catenin pathway in human nasal epithelial cells [80] and in colon cancer cells [81,82]. The expression of S100A4 in myocardial stromal fibroblasts [83] and S100A7 in human epidermal keratinocytes [84] is regulated by TNF-α.

Based on these findings, it appears highly plausible that the expression of certain S100A genes is regulated via the signaling pathways activated by HCSCs or is co-regulated in parallel with other involved genes. As already indicated, specific mechanistic details cannot yet be inferred and should be addressed in future investigations. However, the results of the present study, in conjunction with existing knowledge on S100A proteins, further support the conclusion that S100A proteins play a particularly important role in the differentiation and mineralization processes associated with VPT and RET interventions in human dental pulp.

When examining the most prominent results regarding single S100A genes, it becomes apparent that S100A8 and -A9 genes were not expressed by hDPSCs, neither prior nor following stimulations. This finding is readily comprehensible and can be attributed to the physiological source of the proteins S100A8 and -A9, which is almost exclusively granulocytes, monocytes, and early stages of macrophages [85]. The two proteins are variable and can act solely as dimers or tetramers and were shown to be overexpressed in inflamed dental pulps [34,37]. When used as capping agents, recombinant proteins of S100A8 and -A9 were able to induce dentine bridge formation in rat molars. A functional peptide derivate of protein S100A8 was even able to induce it in the same amount and quality like MTA [39].

Comparable properties were demonstrated for protein S100A7 [38,39]. In our study, lowest concentration of ProRoot^®^MTA led to significant increase in S100A7 gene expression after 72 h, while IL1β did after 24 and 72 h. Protein S100A7 has also been shown to stimulate the differentiation and activity of osteoclasts [40]. It therefore plays a central role in the processes of defense, differentiation, and regeneration of the dental pulp.

Another interesting member of the S100 protein family is S100A4. In addition to its occurrence in PDL cells [86], it has been shown to be highly expressed in odontoblasts and odontblast-like cells, but not in other pulp cells. It therefore appears to be a suitable marker protein for odontoblast and odontoblast-like cells [41,87]. In our study, its expression was upregulated after 72 h following stimulation with the lowest concentration of Biodentine^®^ as well as with IL1β, while other concentrations of Biodentine^®^ or ProRoot^®^MTA had no significant effect. When these results are compared with DSPP expression, another marker of odontogenic differentiation, it becomes evident that, in contrast to S100A4, DSPP was significantly upregulated not only at the lowest concentration of Biodentine^®^, but also at the two lowest concentrations of ProRoot^®^MTA (Figure 4d). The specific function of S100A4 in odontoblasts remains to be elucidated. As this protein is a key player in bone metabolism [88,89], and due to its robust expression in odontoblasts and odontoblast-like cells, it is reasonable to assume that it also plays an important role in the regulation of mineralization processes in these cells. Recently, it was demonstrated to be accumulated at the border area of pulpal calcifications in normal an inflamed human pulp specimens [35].

## 5. Conclusions

In summary, it can be concluded that the two commonly used hydraulic calcium silicate cements ProRoot^®^MTA and Biodentine^®^ have a significant influence on the expression of several S100A genes in human dental pulp stem cells. These results further support the finding that S100A proteins are critically involved in inflammation, differentiation, and mineralization processes in human dental pulp. This is of particular relevance to the pulpal response following interventions performed as part of vital pulp therapy and regenerative endodontic treatments. Further research, including targeted knockdown experiments and pathway-blocking approaches, is needed to clarify the specific functions of the respective S100A proteins.

## Figures and Tables

**Table 1:** Relative differential gene expression level of S100A genes following stimulation with Biodentine^®^ (BD), ProRoot^®^MTA (MTA), IL-1β, and Dexamethasone (Dexa) for 24 h and 72 h. Values are x-fold elevations (marked red) or reductions (marked green) of differential gene expression in relation to unstimulated control cells. Asterisks indicate statistically significant difference at 5% level (*p* < 0.05). Experiments were performed with cells from 2 donors, each in triplicate (*n* = 6 technical replicates). n.d. = not detectable.

24 h		**BD 0.002 mg/mL**				**BD 0.02 mg/mL**				**BD 0.2 mg/mL**				**MTA 0.002 mg/mL**				**MTA 0.02 mg/mL**				**MTA 0.2 mg/mL**				**IL1β 10 ng/mL**				**Dexa 100 nM**			
	Gene	Mean		SD		Mean		SD		Mean		SD		Mean		SD		Mean		SD		Mean		SD		Mean		SD		Mean		SD	
	S100A1	0.64	±	0.2	*	1.15	±	0.5		1.44	±	0.4	*	0.80	±	0.2	*	0.89	±	0.5		0.61	±	0.3	*	1.52	±	0.4	*	0.68	±	0.2	*
	S100A2	0.87	±	0.3		1.08	±	0.5		0.96	±	0.4		0.51	±	0.1	*	0.73	±	0.3		0.73	±	0.2	*	2.16	±	1.1	*	0.49	±	0.1	*
	S100A3	0.63	±	0.1	*	1.13	±	0.5		1.19	±	0.4		0.53	±	0.1	*	0.72	±	0.2	*	0.67	±	0.2	*	2.64	±	1.0	*	0.75	±	0.2	*
	S100A4	0.80	±	0.3		1.00	±	0.4		0.89	±	0.3		0.64	±	0.3	*	0.72	±	0.3		0.69	±	0.3		1.84	±	1.1		0.59	±	0.1	*
	S100A6	1.28	±	0.7		1.49	±	0.7		1.10	±	0.4		0.78	±	0.2		1.00	±	0.4		0.91	±	0.3		2.03	±	1.4		0.77	±	0.3	
	S100A7	0.68	±	0.1	*	1.47	±	0.8		0.76	±	0.7		0.84	±	0.4		0.64	±	0.4		0.59	±	0.5		1.92	±	0.8	*	1.05	±	0.7	
	S100A8		n.d.				n.d.				n.d.				n.d.				n.d.				n.d.				n.d.				n.d.		
	S100A9		n.d.				n.d.				n.d.				n.d.				n.d.				n.d.				n.d.				n.d.		
	S100A10	0.68	±	0.4		1.06	±	0.5		0.86	±	0.3		0.65	±	0.1	*	0.69	±	0.2	*	0.74	±	0.2		1.53	±	0.7		1.02	±	0.3	
	S100A11	0.83	±	0.2		1.02	±	0.4		0.96	±	0.2		0.56	±	0.1	*	0.74	±	0.2	*	0.71	±	0.2	*	2.10	±	0.8	*	0.64	±	0.1	*
	S100A13	0.97	±	0.4		1.11	±	0.5		0.97	±	0.3		0.61	±	0.1	*	0.85	±	0.4		0.77	±	0.2	*	1.63	±	0.9		0.79	±	0.2	
	S100A14	0.79	±	0.6		1.50	±	1.4		0.65	±	0.6		0.32	±	0.2	*	1.19	±	0.7		1.02	±	0.8		0.81	±	0.7		0.90	±	1.1	
	S100A16	0.67	±	0.1	*	1.07	±	0.5		1.15	±	0.4		0.54	±	0.1	*	0.73	±	0.1	*	0.78	±	0.2	*	1.56	±	0.3	*	0.58	±	0.1	*
72 h		**BD 0.002 mg/mL**				**BD 0.02 mg/mL**				**BD 0.2 mg/mL**				**MTA 0.002 mg/mL**				**MTA 0.02 mg/mL**				**MTA 0.2 mg/mL**				**IL1β 10 ng/mL**				**Dexa 100 nM**			
	Gene	Mean		SD		Mean		SD		Mean		SD		Mean		SD		Mean		SD		Mean		SD		Mean		SD		Mean		SD	
	S100A1	1.21	±	0.4		1.28	±	0.4		0.79	±	0.3		0.70	±	0.3		0.59	±	0.3	*	1.38	±	0.5		1.46	±	0.8		0.50	±	0.2	*
	S100A2	1.68	±	0.5	*	1.28	±	0.4		0.91	±	0.2		1.26	±	0.6		0.83	±	0.3		2.03	±	0.8	*	1.66	±	0.3	*	0.78	±	0.4	
	S100A3	1.24	±	0.3		1.17	±	0.3		0.95	±	0.2		1.09	±	0.3		0.80	±	0.3		1.66	±	0.5	*	1.66	±	0.1	*	1.06	±	0.5	
	S100A4	1.74	±	0.3	*	1.35	±	0.3		1.19	±	0.3		1.09	±	0.6		1.09	±	0.5		1.29	±	0.4		2.24	±	0.8	*	0.93	±	0.5	
	S100A6	1.22	±	0.1		1.10	±	0.3		1.04	±	0.2		0.94	±	0.3		1.04	±	0.4		1.17	±	0.3		1.61	±	0.4	*	0.74	±	0.3	
	S100A7	2.16	±	1.6		2.11	±	1.5		0.86	±	0.2		2.25	±	0.8	*	1.06	±	0.6		1.25	±	0.8		2.11	±	1.1	*	1.06	±	0.6	
	S100A8		n.d.				n.d.				n.d.				n.d.				n.d.				n.d.				n.d.				n.d.		
	S100A9		n.d.				n.d.				n.d.				n.d.				n.d.				n.d.				n.d.				n.d.		
	S100A10	1.20	±	0.3		1.01	±	0.3		1.09	±	0.2		0.82	±	0.2		0.78	±	0.2		1.41	±	0.4	*	1.08	±	0.4		1.39	±	0.1	*
	S100A11	1.26	±	0.2		1.14	±	0.3		0.95	±	0.1		1.00	±	0.3		0.74	±	0.2		1.88	±	0.6	*	1.41	±	0.3	*	0.98	±	0.3	
	S100A13	1.53	±	0.4	*	1.27	±	0.4		1.02	±	0.2		1.15	±	0.4		0.79	±	0.2		1.90	±	0.5	*	1.58	±	0.4	*	1.03	±	0.5	
	S100A14	1.37	±	1.5		0.90	±	0.3		0.71	±	0.4		0.55	±	0.1	*	0.56	±	0.4	*	1.04	±	0.6		1.28	±	0.8		0.41	±	0.2	*
	S100A16	1.18	±	0.3		1.15	±	0.3		0.98	±	0.2		1.21	±	0.3		0.87	±	0.1		2.20	±	0.7	*	1.21	±	0.1		0.87	±	0.3	

## Data Availability

The data presented in this study are available on request from the corresponding author.

## Abbreviations

The following abbreviations are used in this manuscript:

ALP	Alkaline Phospatase
ANOVA	Analysis of Variance
ARS	Alizarin Red Staining
CPC	Cetylpyridinium Chloride
DAPI	4′,6-diamidino-2-phenylindole
DMEM	Dulbeccos Modified Eagle’s Medium
DSPP	Dental Sialophosphoprotein
FBS	Fetal Bovine Serum
FGF	Fibroblast Groth Factor
HCSC	Hydraulic Calcium Silicate Cement
hDPC	Human Dental Pulp Cell
hDPSC	Human Dental Pulp Stem Cell
IL	Interleukin
LTA	Lipoteichoic Acid
MTA	Mineral Trioxid Aggregate
PBS	Phsophate Buffered Saline
qPCR	Reverse
RET	Regenerative Endodontic Therapy
RT	Room Temperature
SCAP	Stem Cells Of The Apical Papilla
TBS	Tris-Buffered Saline
VPT	Vital Pulp Therapy
